# Lack of association between six-minute walk distance and pulmonary artery pressure in patients with systemic sclerosis and cardiopulmonary symptoms

**DOI:** 10.34172/jcvtr.2020.42

**Published:** 2020-08-02

**Authors:** Leila Namvar, Alireza Khabbazi, Sabbah Hasani, Masoud Nazemiyeh

**Affiliations:** ^1^Tuberculosis and Lung Disease Research Center, Tabriz University of Medical Sciences, Tabriz, Iran; ^2^Connective Tissue Diseases Research Center, Tabriz University of Medical Sciences, Tabriz, Iran

**Keywords:** Systemic Sclerosis, Six-Minute Walk Test, Six-Minute Walk Distance, Pulmonary Pressure, Pulmonary Hypertension

## Abstract

The six-minute walk test (6MWT) is a non-invasive test used to assess cardiopulmonary performance. The aim of this study was to evaluate the performance of 6MWT in predicting pulmonary artery hypertension (PAH) and interstitial lung disease in patients with systemic sclerosis (SSc) and cardiopulmonary symptoms. Sixty-three patients with SSc who had dyspnea, cough, chest pain and syncope underwent 6MWT, high-resolution computed tomography (HRCT), spirometry, body plethysmography and single breath carbon monoxide diffusion measurement. There were no significant differences in mean 6MWD between patients with diffuse SSc compared with limited disease, patients with no parenchymal involvement compared with patients with parenchymal involvement <20% and≥20% in HRCT, and patients with PAP ≥25 mm Hg compared with patents with PAP <25 mm Hg. No significant relationship was found between 6MWD and age, mean PAP, forced expiratory volume, forced vital capacity and diffusing capacity of the lungs for carbon monoxide. The present study showed that in patients with SSc and cardiopulmonary symptoms, 6MVT does not help to predict PAH and parenchymal lung involvement.

## Dear Editor,

Pulmonary involvement is the most common cause of death in systemic sclerosis (SSc).^1^ The most common pulmonary manifestations of SSc include pulmonary fibrosis and pulmonary artery hypertension (PAH).^1^ The six-minute walk test (6MWT) is a non-invasive test used to assess cardiopulmonary performance in heart and lung diseases.^1^ The aim of this study was to evaluate the performance of 6MWT in predicting PAH and interstitial lung disease in patients with SSc and cardiopulmonary symptoms. In this study 63 patients with SSc who had dyspnea, cough, chest pain and syncope were enrolled (Table 1). Exclusion criteria were, severe anemia (hemoglobin<10 mg/dL), renal failure, Body Mass Index>35), history of pulmonary thromboembolism, chronic obstructive pulmonary disease, valvular heart diseases and heart failure. Studied patients underwent 6MWT, high-resolution computed tomography (HRCT), spirometry, body plethysmography and single breath carbon monoxide diffusion measurement in respiratory lab. The predicted six-minute walk distance (6MWD) for men and women was calculated based on the following formulas.^2^

**Table 1:** Baseline characteristics of participants (N=63)

**Parameters**	
Age mean (SD), years	46.65±9.8
Female (%)	46 (92)
Diffuse SSC (%)	48 (76.2)
Limited SSC (%)	15 (23.8)
Anti-Scl 70 antibody (%)	34 (54)
Anti-Centromere antibody (%)	12 (17.5)
6MWD, m (IQR)	429 (137-727)
FVC % mean (SD)	88.12±11.4
FEV1 % mean (SD)	87.71±11.3
DLCO % mean (SD)	67.01±10.3
PAP mean (SD), mm Hg	33.31±13.1

SSC, systemic sclerosis; SD, standard deviation; IQR, interquartile range; FVC, forced vital capacity; FEV1, forced expiratory volume; DLCO, diffusing capacity of the lungs for carbon monoxide; PAP, pulmonary artery pressure.

*6MWD in men: (7.57×Height) - (5.02×Age) - (1.76 × Weight) - 309 m*

*6MWD in women: (2.11 × Height) - (2.29 × Weight) - (5.78 × Age) + 667 m*

Based on the extent of the lung parenchyma involvement in HRCT, patients were divided into two groups of <20% and ≥20% lung parenchyma involvement. Mean pulmonary arterial pressure (PAP) was measured by echocardiography. Mean PAP≥25 mm Hg was classified abnormal.

Data was analyzed using SPSS 16 software. Categorical variables were expressed as number and percentage. Continuous variables were reported as mean ± SD and for out of range data by median and interquartile range (IQR). Spearman test were used for determination of the relationship between these variables.

**Figure 1:**
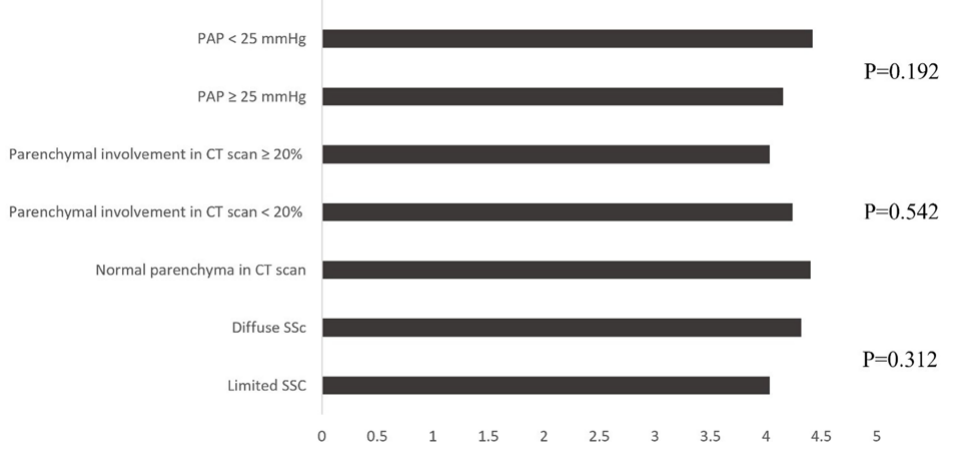
Comparison of 6MWD between the SSc subgroups. Mean PAP, pulmonary artery pressure; CT, computed tomography; SSC, systemic sclerosis

Median 6MWD was 429 meter, which is lower than the reported values for normal subjects (571 ± 90 meters).^2^ In Deuschle et al study 6MWD in SSc patients was 491 meters.^3^ There were no significant differences in mean 6MWD between the SSc subgroups including patients with diffuse SSc compared with limited disease, patients with no parenchymal involvement compared with patients with parenchymal involvement <20% and ≥20% in HRCT, and patients with mPAP ≥25 mmHg compared with patients with PAP <25 mm Hg (Figure 1). No significant relationship was found between 6MWD and age (r=-0.058; P = 0.654), PAP (r=-0.054; *P* = 0.679), FEV1 (r=0.246; *P* = 0.341), FVC (r=0.123; *P* = 0.352) and DLCO (r=-0.088; *P* = 0.542). In contrast to our results, Deuschle et al found a weak to moderate association between 6MWD and pulmonary function parameters such as FVC, FEV1, total lung capacity and DLCO in patients with SSC.^3^ Villalba et al found an association between 6MWD with age, degree of dyspnea and oxygenation in patients with SSc, but the association between 6MWD and FVC was not significant.^4^ They reported no association between 6MWD and pulmonary fibrosis on HRCT. In Sanges et al study 6MWD was significantly related to PAP, but the pulmonary fibrosis did not have a significant impact on 6MWD, indicating that vascular involvement may play more prominent role in limiting functional status.^5^

The walked distance in 6MWT depends on many factors such as patient age, weight, mood, musculoskeletal performance, lung volumes, DLCO, extent of vascular involvement and mean PAP.^6^ SSc is a systemic disease which widely involve skin, musculoskeletal system andhematologic system. Low 6MWD in SSC in addition to cardiopulmonary involvement may be related to chronic anemia, muscle weakness, arthralgia, arthritis and depression. The lack of association between 6MWD with mean PAP and pulmonary function tests in studied patients may be related to the confounding factors mentioned above.

The present study showed that in patients with SSC and cardiopulmonary symptoms, 6MWT does not help to predict PAH and parenchymal lung involvement.

## Competing interests

None declared.

## Acknowledgments

This work was sponsored by a grant from the Deputy of Research of Tabriz University of Medical Sciences. The authors are thankful to our collaborators for their support.

## Ethical approval

The study was designed according to the Helsinki humanity research declaration (2008).

## Funding

None.
